# Age, Allostatic Load, Residential Setting, and Self-Reported Diet Choices Among Older Poles

**DOI:** 10.3390/nu18132095

**Published:** 2026-06-26

**Authors:** Douglas E. Crews, Jan Jeszka, Tatsuya Koyama, Yoshiaki Sone

**Affiliations:** 1 Smith Laboratory, Department of Anthropology, School of Public Health, The Ohio State University, 174 W. 18Th Avenue, Columbus, OH 43210-1106, USA; 2 Department of Human Nutrition and Hygiene, Poznań University of Life Sciences, Wojska Polskiego 31, 60-624 Poznan, Poland; 3 National Institutes of Biomedical Innovation, Health and Nutrition, National Institute of Health and Nutrition Center for Nutritional Epidemiology and Policy Research, Laboratory of Nutrition Guidelines, Kento Innovation Park, NK Building, 3-17 Senrioka Shinmachi, Osaka 566-0002, Japan; t-koyama@nibn.go.jp; 4 Graduate School of Human Life Science, Mimasaka University, 50 Kitazonocho, Okayama 708-8511, Japan; soneyoshi@gmail.com; 5 Osaka City University, 3-3-138 Sugimoto, Sumiyoshi-ku, Osaka 558-858, Japan

**Keywords:** age, allostatic load, diet, Poland, SENECA-method, stress

## Abstract

**Background:** During life, all organisms experience multiple stressful events capable of disrupting their somatic integrity. As mammals, humans respond to environmental, sociocultural, and cognitive stressors via allostasis, a systemic neurophysiological response that supports physiological homeostasis. Unfortunately, allostatic mechanisms are incapable of countering all stressors, and systemic physiological damage accumulates with age; thereby contributing to physiological dysregulation and an increasing allostatic load (AL). Previously, we reported that a ten-factor allostatic load index (ALI) varied significantly by age, gender, and rural–urban residence in a sample of Polish citizens ages 55+ years but a five-biomarker frailty index did not. Here we determine whether an estimated ALI covaries with self-reported food intakes across age, residential setting, and gender. **Methods:** Two hundred and ten residents of Greater Poland ages 55–91 years, residing in either the Nekla commune (N = 103) or the capital of Greater Poland, Poznan (N = 107), participated in research designed to estimate a study-specific 10-biomarker ALI and its possible associations with their self-reported dietary choices, age, gender, and residential location. Of these, 206 completed study protocols including a food frequency questionnaire, verifying their age, self-reporting their gender, and allowing research personnel to obtain data for assessing 10 physiological biomarkers of allostatic load for inclusion in a study-specific ALI. Statistical significance for nominal measures was estimated using chi-square analyses; those for continuous measures, *t*-tests. **Results:** In the full sample, self-reported higher red meat and snack intakes were significantly associated with higher ALI at younger ages (55–69 years). No food item was significantly associated with estimated ALI at older ages (70+ years). Further, low self-reported intakes of fish and seafood consumption were significantly associated with a higher ALI in Poznan, but not Nekla residents. Within the full sample, average ALI was almost identical between younger and older women. **Conclusions:** In this cross-sectional sample of older Nekla and Poznan residents allostatic load not only varied by age, sex, and residential location, but also with self-reported consumption of red meat, snacks, fish and seafood. Observed differences in biomarkers of AL between younger and older residents of Poland across this sample suggest possible higher incidences of chronic disease occur among women residing in Nekla than those in Poznan. Similarly, the significant associations of red meat and snack consumption with ALI at younger ages in both settings may portend increasing vascular disease and related complications among those ages 55–69 years in this sample as they age. As does the higher estimated ALI among Poznan residents reporting low fish and seafood consumption.

## 1. Introduction

To maintain homeostasis, survive, and reproduce, all lifeforms must overcome multiple environmental, e.g., cold, heat, hypoxia, solar radiation, and socioecological stressors, e.g., food and water shortages, intra- and interspecies conflict, predation. Mammals, including humans, do so via an evolved, systemic, somawide neurophysiological stressor abatement system, allostasis [1,2]. Allostatic systems adjust internal physiological activity, e.g., blood pressure, oxygen saturation, circulating cortisol, digestion, heartrate in response to anticipated and experienced stressors. By mitigating stressor-related somatic damage, allostasis supports survival, somatic integrity, and maintenance of a dynamic, yet relatively constant, internal physiological state [1,2,3,4,5,6]. Over their lifespans, humans experience many minor, major, and cognitive stressors capable of initiating a defensive allostatic response. Allostatic response can be physiologically costly and cannot halt all stressor-related damage, nor support somatic systems indefinitely. As we age, our allostatic responsiveness declines, systemic physiological dysfunction progresses, and somatic wear and tear increases, contributing to increasing physiological dysregulation, an allostatic load (AL), and increased risks for chronic illnesses [1,2,3,4,5,6,7,8,9,10,11,12].

One’s exact AL is not directly measurable. However, biomarkers of stressor response (allostasis) are. As are the multiple systemic losses occurring secondary to stressors and allostatic responsiveness itself. Thus, most often an individual’s AL is estimated by whether specific physiological biomarkers related to systemic regulation are dysregulated, e.g., above or below either study-specific estimates, commonly the highest or lowest quartile associated with greatest physiological risk within the sample or based on standard clinical cut points (see [5,6,7,8,9,10,11]). Combining 10 biomarkers of neurophysiological and somatic variation assessed in the McArthur Studies of Successful Aging, Seeman and colleagues developed the first allostatic load index (ALI) [11]. It included 10 physiological assessments associated with human physiological arousal, stress response, morbidity and mortality risks across multiple samples into a single estimate of physiological dysregulation.

Since Seeman and colleagues’ seminal paper [11], a range of study-specific allostatic load indices have been explored and reported for multiple populations as research on allostatic load expanded across health care (see [6,7,8,9,10,11,12,13,14,15,16,17,18]), including explorations of how allostatic load associates with individual and population dietary patterns, self-reported food intakes, preferred food choices, and diet quality. For example, applying a nine biomarker ALI to data from the National Health and Nutrition Examination Survey (NHANES, 2015–2018) among those aged 31 years and older, Zhou and colleagues [13] observed a significant inverse relationship between diet quality and the odds of having an elevated ALI at higher quintiles of the Healthy Eating Index 2015. Similarly, Zhang and colleagues [14] reported a significant association between higher intakes of fruits and whole grains and lower intakes of salt, saturated fats, and sugar, with lower ALI in the NHANES 2015–2018 cohort. Examining compliance with the Dietary Approaches to Stop Hypertension (DASH) among 1565 participants in the NHANES 2017–2020 cohort, Li and LI [15] observed a higher DASH score was significantly associated with a lower 11-biomarker ALI, suggesting a low DASH score increases risk for a higher ALI.

Among older Puerto Rican adults, Mattei and colleagues [16] observed allostatic load was significantly associated with high self-reported meat, processed meat, and French fry consumption. Interestingly for the research reported here, in a cross-sectional survey of Polish adults, Gajda and colleagues [17] observed high-quality healthy diets were more frequently reported by participants residing in small cities (OR = 2.25), but less so among those living in larger cities (OR = 1.67) compared to the baseline for residents of more rural areas. They suggested urban residents may be more informed about healthy eating habits or have more access to healthy eating programs and foods. Conversely, more rural living elders may experience lower quality diets secondary to fewer opportunities to purchase healthy and unprocessed foods. Gajda et al. [17] suggested population characteristics, male gender, rural residence, and low socioeconomic status contribute to poorer diet quality in rural settings, while female gender, urban residence, and higher socioeconomic status may promote better diet quality in more urban settings. Supporting this suggestion, Lipowicz and colleagues [18] observed that higher education, being married, and residing in an urban location were significantly associated with less physiological dysregulation. Further, when unhealthy lifestyle variables were introduced into their model, associations between indicators of SES and allostatic load remained significant, while smoking and alcohol consumption showed little association with SES or allostatic load. In general, physical activity, higher SES, education, and income tend to be significantly associated with less physiological stress and a lower allostatic load across population settings.

Previously we reported men residing in Nekla, Poland, a rural commune, showed a significantly higher average ALI than did those in Poznan, Poland, the capital city of Greater Poland [12]. Among women, ALI was slightly higher although not significantly so in Nekla women than Poznan women. However, based on multivariate regression, ALI did not differ significantly by age or residential location. Additionally, estimated allostatic load did not significantly differ between those aged 55–69 or 70-plus years residing in either Poznan or Nekla [12].

## 2. Methods and Materials

During the summer of 2013, we recruited 216 community volunteers residing in either Nekla or Poznan, Poland to participate in research on their estimated allostatic load index and their self-reported average monthly intakes of 14 standard food items. Using a cross-sectional research design, participants were stratified by age, gender, and residential setting to determine associations of these demographic differences with a 10-item allostatic load index [12]. In both communities, we recruited healthy ambulatory residents who were able to participate in research activities at one of our community study sites, in Nekla at the local Health Clinic and in Poznan at an adult activity center. This sample includes ambulatory Polish citizens aged 55 years and over residing in either Poznan, capital of Greater Poland and its largest city, or Nekla, a small rural commune, including the town of Nekla and several smaller surrounding communities wherein residents participate in a mix of modern and traditional occupational activities. Although differing in their daily lifeways, residents of Nekla generally enjoy the same access to modern cultural conveniences, electricity, air conditioners, automobiles, cellphones, and televisions as those residing in Poznan. Some Nekla residents also commute regularly to occupations in Poznan, others farm and herd, or engage in local manufacturing and commercial pursuits, while also retaining multiple aspects of their ancestors’ livelihoods while enjoying more rural lifeways [12].

Here we explore variation between Nekla and Poznan in biomarkers of allostatic load and whether differences in their reported food choices and serving sizes between these two settings are associated with a 10-biomarker ALI. To determine differences in types and quantities of foods regularly consumed by individuals residing in either Poznan or Nekla, each participant completed a validated self-report dietary history, Survey Europe on Nutrition in the Elderly: A Concerted Action, SENECA-method [19,20]. Responses to the food frequency questionnaire were coded following the Eurocode Food Coding System [21] which includes 13 predefined food types. Associations of individual self-reported food intake portions with the study-specific 10-biomarker ALI were then examined by residential setting (urban Poznan—rural Nekla), age group (ages 55–69 years and 70+ years), and gender. We hypothesized that in a self-selected cross-sectional sample of 216 Polish citizens ages 55 years and older, a 10-biomarker allostatic load index would covary significantly with their self-reported intakes of specific foods identified in the Eurocode Food Coding System.

Inclusion criteria were being ages 55 years or older, residing in one of the two communities selected for research, and being sufficiently healthy and ambulatory to participate in research protocols at their appointed research site. Overall, 216 individuals agreed to participate in study protocols, 126 female and 90 male, among these 110 were aged 55–69 and 106 were aged ≥70 years old (Table 1). Of those assessed, 6 are not included in data analyses due to missing data on one or more biomarkers included in estimating an ALI. Thus, the analytic sample includes 210 individuals (Table 1).

All person-centered research activities: anthropometry, phlebotomy, blood pressure physical assessments, diet and activity questionnaires were completed within the local communities, in Nekla at the Nekla Cultural and Medical Centre Clinic, and in Poznan at the Centre for Geriatrics and Gerontology. All laboratory analyses were completed in the Department of Human Nutrition and Hygiene, Poznan University following international laboratory standards under the direction of Jan Jeszka [12]. Study protocols were conducted following guidelines of the Helsinki Declaration, and all participants provided informed consent prior to participating in any research protocols. All research procedures involving human participants were preapproved by the University of Poznan Research Ethics Committee (Number 425/13).

### 2.1. Research Locations

Poznan was chosen for an urban research location as it is the fifth largest city in Poland by population, the capital of Wielkopolski (Greater Poland), the site of Poznan University, and a major historic region wherein the modern Polish state arose. Modern Poznan includes an area of 261.91 km^2^ making it the seventh largest city by area in Poland, with a population of about 550,742 persons, and a population density of about 2100 persons/km^2^. In Poznan our research was conducted in collaboration with the Centre for Geriatrics and Gerontology. Nekla commune, situated 35 km east of Poznan, was chosen as a less urban research setting, it encompasses only 19.8 km^2^ including the town of Nekla wherein our research occurred and several smaller outlying communities (see [12]). During this research the estimated population of the Nekla commune was 7191 individuals with about 3599 living in the town of Nekla and 3592 residing in smaller villages and outlying farms. Population density within the Nekla commune was about 75 persons/km^2^: in Nekla itself, about 182 person/km^2^, and in outlying villages and regions 47 person/km^2^. About 64% of all land within the Nekla commune is agricultural with 34% forested. The Nekla region has a long history of habitation, being the site of the 10–11th century medieval archeological site Giecz. We gratefully acknowledge and thank all 216 volunteers from Poznan and Nekla who participated in this research.

### 2.2. Materials and Methods

Following a socioecological model, our purpose was to determine if participants’ ALI varied significantly by their gender, socioecological setting, age, self-reported food choices and quantities consumed per day, among those residing in a relatively rural and a more urban setting. To do so, we estimated a 10-biomarker ALI (Table 2) among healthy and mobile residents ages 55 and older residing in Poznan and Nekla. To obtain an appropriate sample for comparison, we recruited participants by posting recruitment flyers at the Nekla Health Clinic and the Centre for Geriatrics and Gerontology. In both settings, we also recruited participants through personal contacts.

During our first meeting with possible participants, we informed them of the study details, likely time commitment, and significance of the project. Those agreeing to participate were advised of the location for their assessments. Either the Nekla Health Clinic or the Centre for Geriatrics and Gerontology, Poznan, provided study details, including their approximate participation time, and were invited to sign an informed consent form, which all did. We then provided all participants with printed and verbal information on why we were conducting this research, along with possible benefits and risks to them and their communities. We requested each to fast for eight hours prior to arriving at their assigned research site and advised all that breakfast would be provided following their blood sampling. We also requested each to complete an overnight urine collection the evening prior to their participation and bring it to their assigned research site the following morning. Of these, three provided inadequate collections and three were inappropriate for proper sampling during laboratory analyses; these six were not included in the final dataset resulting in a total sample of 210 study participants. Prior to their exam day, detailed instructions and a container for urine collection were provided to each participant. They also were provided with phone numbers to contact our research team members [12].

Research protocols included self-reports of health, social and physical activities using a questionnaire provided in either Polish or English; provision of a container for their overnight urine collection; drawing of a 10 mL fasting blood sample; and assessments of walking speed over 50 feet and grip strength using a dynamometer (see [12]). Walking speed over 50 feet and hand grip strength provide direct assessments of mobility and strength at all ages, particularly among elders, and often are included in health surveys and estimates of frailty [12,22]. After having fasted overnight, participants were invited to arrive between 6 and 8 am at their local testing center. Upon arrival each was greeted by a research team member who collected and measured their overnight urine volume before aliquoting a sample for later laboratory analyses. Next, a phlebotomist obtained a 10 mL fasting blood sample and placed it in a refrigerator until it was transported to Poznan University Laboratory Diagnostyka. Following participants’ phlebotomy, they and any accompanying persons, were invited to break their overnight fast at a breakfast buffet including coffee, tea, juices, pastries, boiled eggs, vegetables, fruit, and local food items. After sitting during breakfast, participants relaxed and talked among themselves before proceeding to complete additional assessments, anthropometry, a dynamometer, a health assessment, and a survey of food types consumed regularly and their frequencies of consumption per day (see [12,19,20,21,22]). Engagement between participants and research personnel averaged about three hours.

To complete research aims, we assessed nine components of physiological functionality and one marker of body morphology to estimate a ten-biomarker ALI. These included diastolic blood pressure (DBP), systolic blood pressure (SBP), low-density (LDL) and high-density lipoprotein (HDL) cholesterol, total cholesterol (T-Chol) and low-density lipoprotein (LDL), used to calculate a T-Chol/LDL ratio, serum dehydroepiandrosterone-sulfate (DHEA-S), glycated hemoglobin (HbA1c) and overnight urinary cortisol levels (CCLs), adrenaline (AD) and urinary noradrenaline (NAD) levels, and waist to hip ratio (WHR) (Table 2). For all biomarkers, except HDL and DHEA, for which risk was defined as the lowest quartile, the highest quartile of the sample distribution was used to define high risk.

Systolic and diastolic blood pressures were measured three times over 5 min. The average of the final two measurements is included in data analysis. Biochemical analyses of blood and urine were completed at the Department of Human Nutrition and Hygiene, Laboratory “Diagnostyka”, Poznan University of Life Sciences under the guidance of Professor Jan Jeszka [12]. All person-centered protocols were completed in the Nekla Community Centre or at the Geriatric Center Poznan.

Using these data and the following methods originally proposed by Seeman and colleagues [11], we estimated a study-specific 10-biomarker ALI. Allostatic load indices based on these and similar biomarkers and analytic techniques continue to be widely applied across epidemiology, eldercare, public health, medicine, human biology, biological anthropology, NHANES, and previous research on Polish samples [12,17,18]. Means, standard deviations, ranges, and quartile cut-points for the 10 biomarkers used to estimate our study-specific ALI are included in Table 2. We did not estimate any additional possible formulations, such as clinically based cut-points or z-scores, to estimate additional study-specific ALIs in this analysis. Across this sample, the average ALI was 2.50 with a standard deviation of 1.62 and a range of 0.00–8.00.

### 2.3. Food Intake

Next, we explored associations between the study-specific estimated ALI and self-reported daily intakes of 14 major food groups estimated as number of cups consumed per week. As part of our research protocols during their assessments and interviews, participants completed a food frequency questionnaire (FFQ) administered by a certified dietitian. The FFQ was derived from a diet history questionnaire validated during the SENECA study (Survey Europe on Nutrition in the Elderly: A Concerted Action) [19]. We applied the Eurocode 2 Food Coding System to determine individual food portions consumed [20]. The FFQ includes 128 individual food items. Frequency of intake for each food item included options ranging from ‘once a month or less’ to ‘4–5 times a day’. Participants also self-reported whether they consumed one of three different portion sizes (small, medium, large) for each item ingested. For analyses, we transformed food frequencies into times per day (Table 3) and multiplied them by the standard portion sizes reported [20] to estimate individual food intakes as servings consumed per day. The category ‘once a month or less’ was discounted in these analyses as it was seldom used by participants. This allowed us to express consumption as servings per day for all food categories in equal units: number of eight-ounce cups consumed per day, including partial cups in units of 2.5, 1.0, 0.7 and so forth (Table 3). To determine possible relationships between self-reported food intakes and estimated ALI, all foods were grouped following the Eurocode 2 Food Coding System [20]. The SENECA system predefines food intake into 14 categories: milk and milk products, eggs, fish and sea food, pulses and nuts, grains, sugar and sweets, snacks, plant fat, animal fat, red meat, poultry, vegetables, fruits, and alcoholic beverages.

## 3. Results

### 3.1. Gender, Age, Location

Across this sample, at both ages 55–69 and 70+ years female and male residents of Nekla and Poznan differed significantly from one another across a majority of the 10 biomarkers combined to estimate an ALI (Table 4). For example, Nekla women ages 55–69 years showed significantly higher urinary cortisol, systolic and diastolic blood pressures than their age-matched Poznan peers, while the latter showed lower waist–hip ratio, DHEAs, and urinary norepinephrine, differing in six of 10 biomarkers (Table 4). Those aged 70+ years exhibited significantly higher systolic and diastolic blood pressures than those in Poznan, along with a higher percent HbA1c, as did younger Nekla women (Table 4). Conversely, older women in Poznan also showed significantly higher DHEAs, urinary norepinephrine, and epinephrine than Nekla women (Table 4).

Among Nekla men ages 55–69 years, average diastolic blood pressure, total cholesterol to high-density lipoprotein ratio, and overnight urinary cortisol levels exceeded those among their Poznan contemporaries. Both systolic and diastolic blood pressures were also higher among older Nekla men, reflecting results observed among Nekla women, while urinary noradrenaline was significantly higher among urban men (Table 5). Poznan men also showed a slightly higher waist–hip ratio than Nekla (Table 5). Overall, across rural/urban settings, men showed fewer significant differences in physiological variation than women (Table 4 and Table 5).

### 3.2. Allostatic Load Index (ALI)

Women ages 55–69 years residing in Nekla commune (ALI 2.2) and Poznan (ALI 2.1) showed the lowest and almost identical ALIs (Table 6). Among men, at ages 55–69 years those in Nekla showed the highest ALI (3.43) of any group sampled, including same age Poznan men (2.67). At ages 70+ years, women and men in Nekla did not differ in their average ALIs (2.88). Conversely, at ages 70+ years men and women from Poznan did differ in estimated ALI, although not significantly so. Among the total Nekla sample, men (3.16) and women (2.30) differed significantly in assessed ALI, while men and women in Poznan did not show significant differences in their average ALI (Table 6).

### 3.3. Food Intake and Allostatic Load

Among the fourteen food types included in SENECA, within the full sample a higher self-reported average number of servings of red meat (N = 197, r = 0.21, *p* = 0.003) or snacks (N = 201, r = 0.22, *p* = 0.001) consumed per day, was significantly associated with higher estimated allostatic load (sample sizes for food intakes differ across food groups as not all participants self-reported food intakes across all categories) (Table 7). In Poznan, allostatic load was significantly higher among those reporting greater consumptions of snacks (N = 100, r = 0.31, *p* = 0.001) and red meat (N = 104, r = 0.19, *p* = 0.05). Conversely, more frequent consumption of fish/sea food was associated with a lower ALI (N = 102, r = −0.22, *p* = 0.03). In Nekla, variable intakes of specific foods were not significantly associated with ALI, although eating more red meat showed a borderline association with ALI (N = 93, r = 0.19, *p* = 0.06). Among participants aged 55–69 years, higher consumption of snacks (N = 103, r = 0.29, *p* < 0.01) and red meat (N = 98, r = 0.24, *p* = 0.02) was significantly associated with higher estimated ALI (Table 7). Among those aged 70+ years no significant associations between intakes of specific foods and the ALI were observed (Table 7). However, both red meat (N = 101, r = 0.19, *p* = 0.06) and poultry (N = 99, r = 0.19, *p* = 0.06) consumption did show borderline significant associations with ALI (Table 7).

To further investigate associations of specific food intakes with allostatic load, we estimated the odds of a participant having an ALI ≥ 3 given their self-reported food intake patterns. Those who reported not eating plant fat (OR 2.98, *p* = 0.03), or fish and seafood (OR 3.38, *p* = 0.02), but eating more than one snack serving per day (OR 2.04, *p* = 0.02), showed higher probabilities of having an allostatic load ≥ 3. Conversely, those reporting greater than 0.3 servings of fish and seafood per day (OR 0.07, *p* = <0.001) or less than 0.5 servings/day of red meat (OR 0.28, *p* = 0.002) showed lower probabilities of attaining an AL ≥ 3.

## 4. Discussion

To explore variability in physiological aspects of health among older residents of Greater Poland residing in varying socioecological settings, we recruited 210 adult participants ranging from 55 to 91 years of age. Of these, 107 resided in Poznan, the capital of Greater Poland, and 103 in Nekla, a rural commune about 35 km southwest of Poznan. Following standard methods for estimating allostatic load indices published across multiple venues [4,5,6,7,8,9,10,11,12,13,14,15,16,17,18], we assessed and combined 10 physiological biomarkers of health (Table 4) into a study-specific allostatic load index. We then compared estimated ALIs across age groups, younger (55–69) and older (ages 70–91), by gender, and between residential locations, Poznan and Nekla. Although multiple physiological biomarkers of allostasis differed significantly across residential settings and gender suggesting variable stressor experiences between them (Table 4 and Table 5), estimated ALIs differed little by gender, residential settings, and age group (Table 6).

### 4.1. Age, Residence, and Gender Differences in Physiological Biomarkers

At ages 55–69 years, women residing in Nekla showed significantly higher urinary cortisol, systolic and diastolic blood pressure, but lower waist–hip ratio, DHEAs, and urinary norepinephrine compared to their age-matched Poznan peers (Table 4). Those aged 70+ years residing in Nekla expressed significantly higher systolic and diastolic blood pressures than their Poznan counterparts, along with a significantly higher percent HbA1c (Table 4), as did younger women. Conversely, women of 70+ years in Poznan showed higher DHEAs, urinary norepinephrine, and epinephrine, possibly reflecting differences in local stressor exposures associated with urban settings.

Among younger but not older participants, waist–hip ratio, and urinary cortisol differed significantly between groups, while among older participants but not younger participants glycated hemoglobin and urinary epinephrine differed significantly (Table 4). Both systolic and diastolic blood pressure were significantly higher among younger than older women residing in Nekla than those in Poznan suggesting differing lifestyles may influence disease risks, health, and survival (Table 4). Younger Nekla women also showed lower average waist–hip ratio, DHEAS, and norepinephrine levels than age-matched Poznan women (Table 4). Although observations of higher systolic and diastolic blood pressures, and cortisol are cross-sectional, they do suggest possible higher risks for chronic conditions among Nekla women in both age groups as they age.

Between Poznan and Nekla, men showed fewer significant differences in their physiological assessments than women (Table 4 and Table 5). At ages 55–69, Poznan men exhibited a significantly higher average waist–hip ratio than Nekla men, who showed higher average diastolic blood pressure, total to HDL cholesterol ratio, and urinary cortisol (Table 5). While older men differed in only three biomarkers of allostatic load, with Nekla men having higher systolic and diastolic blood pressures, while Poznan men showed higher urinary norepinephrine (Table 5). Results of this cross-sectional research among Nekla men suggest possible greater risks for cardiovascular chronic conditions among men from Nekla than from Poznan, as did results among women. Older men also differed in several biomarkers of allostatic load between settings, but fewer than women. Those in Nekla showed higher systolic and diastolic blood pressures than Poznan men, while Poznan men exhibited higher urinary norepinephrine (Table 5), as did younger men. As observed among women, these results suggest men living in Nekla may experience higher risks for chronic conditions than those in Poznan.

### 4.2. Allostatic Load, Age, Sex, and Residence

Within this sample estimated allostatic load differed little by age, sex, or residence. Only between all Nekla men and women were significant differences in estimated ALI observed (Table 6). In the full samples of Nekla men and women, men showed a significantly higher estimated ALI (3.16) compared to women (2.30). At ages 55–69 years, younger Nekla men also showed a higher ALI (3.43) than women (2.22). At ages 55–69 years, women residing in both Nekla (ALI 2.2) and Poznan (ALI 2.1) showed the lowest and almost identical ALIs (Table 6), while men in both showed the highest. At ages 70+, women and men in Nekla shared the same estimated ALI (2.88), although the range among women exceeded that of men.

### 4.3. Self-Reported Food Choices and Allostatic Load

Research protocols included requesting all participants to complete a self-report food frequency questionnaire (FFQ). These allowed us to explore associations of their food choices with their estimated ALI by age, gender, and rural–urban location. Higher self-reported consumption of red meat and snacks were observed to associate significantly with ALI among younger, but not older participants. Among older residents of Poznan, but not Nekla, greater self-reported snack and lower fish and seafood consumption both were significantly associated with a higher observed ALI. Overall, most food groups examined were not significantly associated with the ALI.

At ages 55–69 years, intake frequencies of specific food groups were significantly associated with ALI in both the total and Poznan samples, but not among Nekla residents. Specifically, more frequent consumption of red meat and snacks, along with low intakes of fish and seafood correlated significantly with higher ALI in Poznan. Conversely, in the full sample higher intakes of fish, other seafood, and plant fats were associated with a lower ALI. These results reflect those of Kynde et al. [20] who observed declines in physical performance in association with higher intakes of carbohydrates and Mattei and colleagues [16] who reported that higher intakes of red meat, processed meat, and French fries were significantly associated with a higher allostatic load index among older Puerto Ricans. They also reflect results from Choi et al. [22] who reported a significant association between greater consumption of fish and a lower allostatic load index in a national sample of older Americans. Results reported here for a sample of older adults residing in Poland align with these previous studies suggesting a broad association of specific dietary components with allostatic load.

## 5. Conclusions

In this sample of Polish residents of Nekla and Poznan, Poland, aged 55–91 years, allostatic load was influenced significantly by age, residential location, and self-reported consumption of red meat, snacks, fish and seafood. However, neither area of residence (urban/rural), nor being older (70+ years) or younger (ages 55–69) were significantly associated with a 10-biomarker ALI among elders residing in Poland’s Wielkopolski Region (Table 6). However, being male and residing in Nekla was significantly associated with a higher ALI than Nekla women and the highest ALI observed in any subsample. Intake frequencies of specific foods also were significantly associated with ALI in both the total sample and urban subgroup aged 55–69 years. Specifically, more frequent consumption of red meat and snacks, along with low intakes of fish and seafood were associated with a higher ALI, while higher intakes of fish and other sea food, along with greater intakes of plant fats were associated with a lower ALI, as with multiple previous allostatic load samples.

## 6. Limitations

During summer 2013 we recruited 210 volunteer research participants aged 65 years old residing either in urban Poznan or more rural Nekla, Poland. The goal for this research was to explore associations of a study-specific 10-biomarker allostatic load index with individual dietary choices, age, gender, and residential settings. The data and results reported here represent a one-time cross-sectional survey among these two communities. Thus, only correlational, not causal associations, may be inferred from these data. Additionally, it is possible that volunteers recruited for this research do not represent the full range of older residents in the participating communities, nor their health status or allostatic load. Finally, the methods for determining individual intakes of specific food groups often are confounded by recall bias and social desirability issues, which may influence the data reported here.

## Figures and Tables

**Table 1:** Age, sex, and residential distribution of all assessed participants.

Age	Sex	Poznan	Nekla	Mean SD
50–69 years	Women	37	29	68.2 (6.8)
	Men	18	26	69.1 (8.5)
70+ years	Women	35	25	70.5 (6.3)
	Men	21	25	70.7 (7.1)
Totals		111	105	69.8 (7.9)

**Table 2:** Means, standard deviations, ranges, and high-risk quartile bound for biomarkers of allostatic load.

Biomarker	Mean	SD	Range	Quartile
Waist–hip ratio	0.889	0.08	0.66–0.108	0.94
Percent HbA1c	6.22	0.75	0.80–10.6	6.4
Dehydroepiandrosterone sulfate, mg/dL	125.4	81.8	15.8–499	66.2
Systolic blood pressure, mmHg	138.8	18.6	97–192	151
Diastolic blood pressure, mmHg	78.4	12.9	7–123	86
HDL cholesterol mg/dL	29.3	23.2	11.1–348	46
Total cholesterol/HDL cholesterol	3.9	0.1	2.1–9.0	4.5
Urinary cortisol, nmol/g creatinine	84.9	59.1	11.4–321	111
Urinary norepinephrine, mg/g creatinine	35.5	15.1	9.6–104	44.2
Urinary epinephrine, mg/g creatinine	6.0	6.2	0.0–35.7	9.3

Note: For HDL and DHEA-s the highest risk occurs in the lowest quartiles, all others in the highest quartile. IBM SPSS Statistics 22 was used for all statistical analyses.

**Table 3:** Transformation of frequency of consumption into homogenous units based on cups consumed per day.

Food Frequency	Times per Day
4–5 times/day	4.5
2–3 times/day	2.5
1 time/day	1.0
4–6 times/week	0.7
2–3 times/week	0.35
1 time/week	0.14
2–3 times/month	0.09

**Table 4:** Urban–rural differences in biomarkers of allostatic load among women by age and location.

Women: Ages 55–69 years							
	Nekla			Poznan			
	Mean	SEM	N	Mean	SEM	N	*p*
Waist–hip ratio	0.82	0.0	29	0.90	0.0	37	s
Percent HbA1c	6.1	0.1	27	6.0	0.1	36	ns
DHEA sulfate, mg/dL	101.2	11.2	28	118.9	9.1	35	s
Systolic bp, mmHg	142	2.8	29	132.4	2.6	34	s
Diastolic bp, mmHg	83	1.8	29	74.2	1.8	34	s
HDL cholesterol mg/dL	58.8	2.2	25	59.7	1.9	35	ns
Total/HDL cholesterol	3.6	0.1	28	3.7	0.1	36	ns
Urinary cortisol, nmol/g c	90.6	8.4	29	75.9	10.3	36	s
Urinary norepinephrine, mg/g c	0.06	0.1	28	0.13	0.02	30	s
Urinary epinephrine, mg/g c	33.3	2.2	28	34.6	1.8	37	ns
Women: Ages 70+ years							
	Nekla			Poznan			
	Mean	SEM	N	Mean	SEM	N	*p*
Waist–hip ratio	0.85	0.0	23	0.8	0.0	35	ns
Percent HbA1c	6.2	0.1	23	5.9	0.1	35	s
DHEA sulfate, mg/dL	81.9	9.4	23	85.6	8.2	34	s
Systolic bp, mmHg	149	3.6	22	135	2.5	34	s
Diastolic bp, mmHg	83	2.9	22	76	1.7	33	s
HDL cholesterol mg/dL	56.8	1.7	25	58.4	2.0	33	ns
Total/HDL cholesterol	3.6	0.2	25	3.7	0.2	34	ns
Urinary cortisol, nmol/g c	82.6	11.6	25	78.4	12.8	35	ns
Urinary norepinephrine, mg/g c	0.04	0.1	25	0.11	0.02	31	s
Urinary epinephrine, mg/g c	37.9	4.0	24	45.3	3.2	35	s

Analyses: Normally distributed via *t*-tests; nonnormal via Mann–Whitney U tests. *p* = 0.05 or less considered statistically significant (s), other values nonsignificant (ns).

**Table 5:** Urban–rural differences in physiological biomarkers of allostatic load by age: men.

Men: Ages 55–69 years							
	Nekla			Poznan			
	Mean	SEM	N	Mean	SEM	N	*p*
Waist–hip ratio	0.94	0.0	26	0.98	0.0	18	s
Percent HbA1c	6.1	0.1	23	6.2	0.1	16	ns
DHEA sulfate, mg/dL	189	15.8	25	165	22.5	17	ns
Systolic bp, mmHg	141	3.5	23	135	2.7	18	ns
Diastolic bp, mmHg	82	2.1	24	74	1.8	18	s
HDL cholesterol mg/dL	46.3	1.8	26	49.1	3.2	18	ns
Total/HDL cholesterol	4.5	0.2	24	3.7	0.2	16	s
Urinary cortisol, nmol/g c	85.5	9.8	26	53.3	9.8	17	s
Urinary norepinephrine, mg/g c	0.09	0.02	25	0.09	0.03	16	ns
Urinary epinephrine, mg/g c	33.6	2.9	26	27.1	3.2	17	ns
Men: Ages 70+ years							
	Nekla			Poznan			
	Mean	SEM	N	Mean	SEM	N	*p*
Waist–hip ratio	0.95	0.0	25	0.93	0.0	21	ns
Percent HbA1c	6.2	0.1	24	6.0	0.1	19	ns
DHEA sulfate, mg/dL	118.3	11.4	24	97.7	9.7	20	ns
Systolic bp, mmHg	153	2.8	24	134	3.1	21	s
Diastolic bp, mmHg	80.0	2.4	25	75	2.2	21	s
HDL cholesterol mg/dL	50.9	2.4	25	47.9	2.0	21	ns
Total/HDL cholesterol	3.8	0.2	25	3.6	0.2	21	ns
Urinary cortisol, nmol/g c	111.2	16.8	23	80.7	10.4	21	ns
Urinary norepinephrine, mg/g c	0.03	0.1	24	0.09	0.02	19	s
Urinary epinephrine, mg/g c	31.1	2.2	23	33.8	2.7	21	ns

Analyses: Normal distributed via *t*-tests; nonnormal via Mann–Whitney U tests. *p* = 0.05 or less considered statistically significant (s), other values nonsignificant (ns).

**Table 6:** Estimated allostatic load index by age, location, and gender N = 210.

	Nekla				Poznan			
Age	Women		Men		Women		Men	
55–69	28	2.20 (1.6–2.8)	26	3.43 (2.7–4.1)	36	2.10 (1.6–2.6)	15	2.67 (1.9–3.5)
70+	25	2.88 (2.5–4.1)	24	2.88 (2.4–3.5)	35	2.30 (1.7–3.0)	21	2.71 (2.0–3.5)
Total	53	2.30 (1.9–2.4)	50	3.16 (2.7–3.2)	71	2.20 (1.8–2.6)	36	2.69 (2.2–3.2)

**Table 7:** Spearman correlation coefficient between food group consumption frequency and ALI.

Food Group	Total			Urban			Rural			55–69 Years			≥70 Years		
	N *	r	*p*	N *	r	*p*	N *	r	*p*	N *	r	*p*	N *	r	*p*
Milk and milk products	195	−0.10	0.18	97	−0.12	0.24	98	−0.03	0.80	99	−0.05	0.62	96	−0.15	0.14
Eggs	201	0.10	0.15	103	0.14	0.17	98	0.08	0.42	101	0.08	0.44	100	0.13	0.20
Fish and sea food	200	−0.10	0.18	102	−0.22	0.03	98	0.01	0.88	99	−0.15	0.14	101	−0.04	0.66
Red meat	197	0.21	0.003	104	0.19	0.05	93	0.19	0.06	98	0.24	0.02	99	0.19	0.06
Poultry	199	−0.02	0.83	102	0.19	0.60	97	0.07	0.52	98	0.16	0.12	101	−0.19	0.06
Grains	200	0.00	0.99	100	0.05	0.64	100	−0.09	0.36	99	0.09	0.40	101	−0.04	0.66
Pulses and nuts	199	0.00	0.98	98	0.02	0.83	101	−0.03	0.76	100	−0.07	0.50	99	0.07	0.50
Vegetables	201	0.07	0.35	101	0.12	0.23	100	0.03	0.78	101	0.14	0.17	100	0.00	0.98
Fruits	197	−0.03	0.72	98	−0.09	0.39	99	0.06	0.59	99	−0.10	0.31	98	0.05	0.61
Plant fat	198	0.00	0.98	100	0.11	0.27	98	−0.08	0.42	97	−0.08	0.41	101	0.08	0.45
Animal fat	200	0.03	0.65	98	0.02	0.87	102	0.01	0.95	99	0.02	0.88	101	0.06	0.58
Snacks	201	0.22	0.001	100	0.31	0.001	98	0.13	0.20	103	0.29	0.001	98	0.15	0.15
Sugar and sweets	198	−0.08	0.27	98	0.02	0.83	100	−0.07	0.51	100	−0.14	0.17	98	−0.01	0.92
Alcoholic beverages	201	0.10	0.14	102	0.09	0.38	99	0.06	0.59	99	0.19	0.06	102	0.03	0.76

* Extreme outliers were excluded from analyses.

## Data Availability

Data included in this manuscript are available from the corresponding author upon reasonable request.
